# Medical Approach to Refugees: Importance of the Caring Physician

**DOI:** 10.5334/aogh.2779

**Published:** 2020-04-16

**Authors:** Patrícia Deps, Philippe Charlier

**Affiliations:** 1 Department of Social Medicine, Federal University of Espírito Santo, Vitória, Espírito Santo, BR; 2 Paris-Saclay University (UVSQ), Laboratory of Anthropology, Archaeology & Biology (LAAB), Montigny-Le-Bretonneux, FR; 3 Department of Research and Higher Education, Musée du quai Branly - Jacques Chirac, Paris, FR

## Abstract

Migrants pose many challenges to recipient countries, including preparedness and resilience of health systems and provision of access to health services. Refugees and asylum seekers comprise a heterogenous group with significant and complex health needs, including the physical and mental consequences of violence. Physicians are often on the frontline in providing care for these unassisted, vulnerable and often maligned and misunderstood population groups. The need to adopt an effective and compassionate medical approach is imperative, including an awareness of unconscious biases and differences in gender roles, decision-making, and social taboos. In addition to practical steps to promote and build a trusting relationship between patient and physician during consultations, physicians require a broad knowledge of anthropology, history, sociology, and drivers of migration such as conflict, resource scarcity and climate change for a better understanding of their patients.

Since humans have organized themselves into societies, beginning in prehistory, they have migrated according to their needs, whether for food, land, shelter, or in flight from predators or natural catastrophes. Sometime later, this movement has had other motives, such as job-seeking, commerce, religious expeditions, or escape from epidemics, conflict, environmental catastrophes, or religious, ethnic, and political persecution [1]. The term ‘refugee’ appeared in 1685 when Protestants (Huguenots) fled France for fear of being persecuted and massacred. In the following centuries, few actions were taken to help and protect migrants until, in 1950, the United Nations High Commission for Refugees (UNHCR) was created to help Europeans displaced by the Second World War.

Refugees and migrants are not the same. A migrant is a person who moves from one country to another to live, whereas the definition of refugee is more complex. A refugee, according to the UNHCR convention, is defined as a person who has a ‘well-founded fear of being persecuted for reasons of race, religion, nationality, membership of a particular social group or political opinion, is outside the country of his/her nationality and is unable or, owing to such fear, is unwilling to avail him/herself of the protection of that country; or who, not having a nationality and being outside the country of his/her former habitual residence as a result of such events, is unable or, owing to such fear, is unwilling to return to it’ [2].

Migration is a complex issue [3], and migratory movements have intensified on all continents in recent decades, mainly driven by wars, territorial disputes, terrorism, environmental changes, extreme poverty, and religious, political, and ethnic conflict. According to UNHCR, in 2018 there were approximately 68.5 million people around the world who needed to leave home and find another place to live. There are about 10 million stateless persons denied nationality and access to fundamental rights such as education, health, employment, and freedom of movement [2]. The five European countries that received the highest number of migrants in 2016 were, in descending order, Germany, Sweden, Hungary, Italy, Austria, and France, with people arriving mainly from the Middle East and Africa [4,5].

Physicians are often on the frontline in assisting refugees, many, but not all, of whom are victims of physical and mental violence. The need to adopt an effective and compassionate medical approach that responds to the complex health needs of displaced populations and addresses the root causes of displacement is therefore imperative [6]. Psychosomatic disorders in migrants are highly prevalent, together with less common disorders such as Ulysses Syndrome or Syndrome of the Extreme Migratory Duel [7,8]. Among asylum seekers who arrived in Denmark and Norway, 45–60% had been subjected to torture [9,10]. Physicians are obliged to document cases of torture, in accordance with the WHO Istanbul Protocol, an international guideline for medical documentation of torture [11]. Other difficulties frequently encountered by refugees include communication, homelessness, lack of work, family alienation, and physical and mental violence inflicted en route to and in the host country [12]. The stressors accompanying migration and the adverse effects of these on physical and mental health, whether visible or invisible, are common to migrating individuals, families, and groups regardless of migrant typology [13].

The growing influx of vulnerable populations poses many challenges to host countries, not least regarding preparedness and resilience of health systems and access to health services [6]. The manifold difficulties experienced by refugees are exacerbated and prolonged when they encounter host country physicians who are unprepared and do not recognize the importance of refugees’ backgrounds in relation to their health [14,15]. Physicians therefore need to acquire skills to provide care for unassisted and often maligned and misunderstood displaced populations. This requires educators and physicians to have the opportunity to develop knowledge around cultural diversity, empathy, and medical humanities, to obtain a better understanding of people seeking assistance [16]. Medical staff providing care to refugees need training in enhanced medical interview techniques to obtain information needed for reliable diagnosis and effective assistance to victims of violence and torture [11].

## Building a Good Patient-physician Relationship

Refugees and asylum seekers comprise a vulnerable and heterogenous group with significant and complex health needs [17]. Medical care should be based on the values of the patients, not on the values of the professionals who provide care. It is important for the physician to recognise they may be influenced by unconscious bias arising from misbeliefs regarding the person with whom they are interacting. Cultural values in refugee groups can manifest as differences in gender roles, decision-making, social taboos, and time-orientation, and these differences can present challenges to the physician-patient relationship [18]. The presence of a same-gender chaperone, usually a relative, social worker, or interpreter, is often necessary.

The medical team should be sensitive to and observe practices that promote and build a trusting relationship between patient and physician. It is essential that the physician reads the patient’s dossier before starting the consultation, including any social and psychosocial notes. A quiet, comfortable consultation environment should be provided with guaranteed privacy. Establish whether the patient needs food or water before starting the interview, consider their whole well-being, and provide or signpost assistance with their wider needs [12]. Refugees who are culturally different from physicians often have names that are difficult to pronounce. It is of critical importance that doctors strive to pronounce the patient’s name correctly and take an active interest in their background, language and culture [12]. Basic good practice, like the doctor introducing her/himself by name, explaining her/his role, and how she/he will try to help the patient are essential measures that engender a secure basis and positive atmosphere to build a trusting doctor-patient relationship.

Having a compassionate and empathetic disposition is important in relationship-building [19]. Patients often feel sad and start crying during the medical interview. It is crucial for the physician to listen and to avoid stereotyped behaviours, to respect the patient’s feelings, and to try to understand the reason for certain attitudes and behaviours and the contextual background to the patient’s story. The medical interview should be structured in an open format, offering the patient freedom to choose where to start and finish their story.

In cases of victims of violence and survivors of torture, the practice of delimiting the motive of the consultation can sometimes be of no benefit. Patients may not readily share their stories by their own initiative [20]. It is essential to allow and encourage the patient to formulate in their own words the history of their physical injuries and traumas related to violence, abuse, and torture. Allow the patients to complete their statements without interruption, except if necessary to clarify patient statements that are unclear or require amplification. However, phrases that reinforce the bonds of trust such as ‘Don’t worry about time,’ ‘Take as much time as you need,’ ‘I will be here with you,’ ‘How can I help you?’ and ‘I will be here to listen to you’ are extremely helpful.

Physicians can help to establish a chronology using a timetable. Explore the patient’s concerns, not only about their physical injuries, but about their family, social, and work situation, using techniques such as facilitation to support the patient’s narrative, demonstrating empathy, and avoiding conscious and unconscious ethical and ideological prejudice and bias. Being non-judgmental is of primary importance. Direct or indirect criticism of refugees’ behaviours should be avoided, using instead supportive narrative techniques such as ‘functional silence’ to open the possibility of accepting/creating moments and ‘acceptance’ of the legitimacy of the patient’s feelings and points of view. At the end of the medical interview, even before the physical examination, patient’s information must be summarized.

It is impossible to ascertain the exact number of those experiencing torture annually, because torture is underreported due to fear, humiliation, shame, lack of support from lawyers and physicians, or risk of deportation and exposure to further harm or death. On current trends, western European physicians will be increasingly confronted with physical and mental health problems related to torture and detention [21]. Lesions resulting from torture are characteristic in many cases. Whilst these tell the victims’ stories, information about the occurrence of injuries and the timing of the causative events and treatment and healing processes may be not accurate because the passage of time may be measured or recollected differently in different cultures. Concomitant physical and mental injuries with behavioural change as occur in cases of post-traumatic stress disorder can make patients tell their history imprecisely. Therefore, cultural knowledge of refugees’ places of origin, as well as methods of torture in specific countries, are invaluable to the physician [22].

Refugees who manage to reach a country that shelters them seek a way to establish themselves legally and to play their role in society. This path is often obstructed by bureaucracy and officialdom, but mainly by a sense of non-belonging. Difficulties encountered in the new society can be immense, although some European countries have established well-organized centres to receive and support refugees and asylum seekers. Practical barriers impeding access by refugees to health services include inadequate information and awareness about the availability of services, insufficient financial means, restricted access to transport, culturally insensitive care, and inadequate provision of interpreters [6,9]. Various countries also charge migrants user fees for health-care services, further restricting access to health care [23].

Physicians practicing in countries with inward migratory movements require a broad knowledge of anthropology, history, sociology, and drivers of migration such as conflict, resource scarcity and climate change for a better understanding of the population to be assisted. It is incumbent on medical schools to include development of empathy and teaching on refugee health within the human and social sciences curriculum, and to further this during foundation medical training. This will prepare a cohort of physicians with the requisite skills and knowledge to provide a good standard of medical care for displaced people.
